# State of the science on postacute rehabilitation: setting a research agenda and developing an evidence base for practice and public policy: an introduction

**DOI:** 10.1186/1743-0003-4-43

**Published:** 2007-11-02

**Authors:** Allen W Heinemann

**Affiliations:** 1 Feinberg School of Medicine, Northwestern University, and Rehabilitation Institute of Chicago, Chicago, IL USA

## Abstract

The Rehabilitation Research and Training Center on Measuring Rehabilitation Outcomes and Effectiveness along with academic, professional, provider, accreditor and other organizations, sponsored a 2-day State-of-the-Science of Post-Acute Rehabilitation Symposium in February 2007. The aim of this symposium was to serve as a catalyst for expanded research on postacute care (PAC) rehabilitation so that health policy is founded on a solid evidence base. The goals were to: (1) describe the state of our knowledge regarding utilization, organization and outcomes of postacute rehabilitation settings, (2) identify methodologic and measurement challenges to conducting research, (3) foster the exchange of ideas among researchers, policymakers, industry representatives, funding agency staff, consumers and advocacy groups, and (4) identify critical questions related to setting, delivery, payment and effectiveness of rehabilitation services. Plenary presentation and state-of-the-science summaries were organized around four themes: (1) the need for improved *measurement *of key rehabilitation variables and *methods *to collect and analyze this information, (2) factors that influence *access *to postacute rehabilitation care, (3) similarities and differences in quality and quantity of services across PAC *settings*, and (4) effectiveness of postacute rehabilitation services. The full set of symposium articles, including recommendations for future research, appear in *Archives of Physical Medicine and Rehabilitation*.

## Background

The growing population of older adults who sustain strokes, hip fractures, joint replacements, and other conditions, Centers for Medicare & Medicaid Services' (CMS) inpatient prospective payment system (PPS), and technical advances in medical and surgical care have led to increasing demand for medical rehabilitation services. Medical rehabilitation provides crucial services that help people with chronic illness and disability learn to live as independently as possible. In inpatient rehabilitation facilities, physician coordinated, multidisciplinary teams focus on reducing impairments, enhancing independence in daily activities and quality of life, and minimizing caregiver burden. As documented in a recent Medicare Payment Advisory Commission (MedPAC) report [1], the health care industry has responded to greater demand by increasing the number of hospital and skilled nursing facility (SNF) beds, and therapists and nurses providing home health services.

Over the past 20 years the cost of postacute services, including postacute rehabilitation services, have grown much faster than overall inflation, reflecting an increased demand for services and growth in number of providers. The U.S. Congress passed a series of laws (eg, Balanced Budget Act of 1997, Balanced Budget Refinement Act of 1999, Deficit Reduction Act of 2005) intended to reduce Medicare's PAC expenditures by establishing and refining PPSs for rehabilitation hospitals, nursing homes, long-term care hospitals (LTCHs), and home health (HHAs).

Changes in payment mechanisms alters providers' incentives and indirectly the organization and availability of PAC. The consequences of payment changes on Medicare beneficiaries' access to high-quality rehabilitation services, independence, and quality of life are unknown. Research on access to, organization of, and the effectiveness of rehabilitation services is needed in order to understand the consequences of new payment mechanisms.

Rehabilitation-focused health services research has concentrated on patients' natural recovery in single types of rehabilitation settings – rehabilitation hospitals and units, SNFs, LTCHs, and HHAs. It is often too expensive and unfeasible to evaluate costs and benefits of rehabilitation across sites of care, let alone specific paths of care, such as from hospitals to nursing homes to home. We know that most patients' functional independence improves during rehabilitation, but we know little about the "active ingredients" of rehabilitation and which types of patients are best suited for which setting so that optimal outcomes are achieved at a reasonable cost.

Comparing outcomes across postacute settings has been hampered by the lack of a common outcome assessment instrument across settings, or a cross-walk between the instruments used by rehabilitation hospitals, SNFs, LTCHs, and home health agencies (HHAs). Imagine if Maryland's weights and measures differed from California's and Illinois's and Texas's – and we had no way to convert their measures. With only a bit of hyperbole, this is the situation Medicare finds itself in when trying to evaluate the relative effectiveness and cost effectiveness of rehabilitation hospitals, nursing homes, LTCHs, and HHAs.

In the absence of scientific evidence and a way to compare outcomes across settings, Medicare has promulgated rules that limit access to IRFs. The so-called "75% rule" and Medicare fiscal intermediaries' "local coverage determinations" are based on expert opinion and on a dearth of scientific evidence. In developing these regulations, Medicare was dependent on anecdotal information. While the 75% rule was written to distinguish rehabilitation hospitals and units from acute care hospitals, Medicare revised inpatient rehabilitation facility (IRF) regulations to require explicit documentation of medical necessity and adopted the 75% rule to limit the types of patients admitted. Beneficiaries' access to rehabilitation services could suffer if the truism that "the absence of evidence of effectiveness does not imply evidence of absence of effectiveness" is not recognized.

The need for expanded rehabilitation-focused health services research was addressed during a workshop in 2005 that was sponsored by the National Center for Medical Rehabilitation and Research (NCMRR) within the National Institute of Child Health and Human Development (NICHD) and the CMS [2]. Participants identified a number of research priorities, including a randomized controlled trial of rehabilitation contrasting inpatient rehabilitation with skilled nursing home rehabilitation for patients with hip fractures. Also identified was the need for research on intensive rehabilitation for patients with major joint replacements, and those with cardiac and pulmonary conditions. Participants also called for studies to better characterize rehabilitation facilities. While director of NICHD, Duane Alexander, MD, promised to seek funding for targeted initiatives, he thought providers might have to provide protected time for investigators to participate in trials and help collect data for such a study, and that providers could conduct their own small population studies without waiting for federal funding. The need for additional research that would inform health policy was stated clearly.

### Symposium planning

The Rehabilitation Research and Training Center on Measuring Rehabilitation Outcomes and Effectiveness, funded by the National Institute on Disability and Rehabilitation Research (NIDRR), was asked to lead the planning for what became the Symposium on Post-Acute Rehabilitation. The symposium was guided by a planning committee (see Acknowledgments) with representatives from the American Academy of Physical Medicine and Rehabilitation, the American Congress of Rehabilitation Medicine, the Association of Academic Physiatrists, the Foundation for Physical Medicine and Rehabilitation, the American Hospital Association, and the Federation for American Hospitals. The same organizations provided financial support. Major financial and staff support was provided by the American Medical Rehabilitation Providers Association (AMRPA). Additional sponsors included the American Physiatric Education Council, CARF International (formerly the Commission on Accreditation of Rehabilitation Facilities), Casa Colina Centers for Rehabilitation, eRehabData, Fowler Healthcare Associates, HealthSouth Corporation, IT Health Track, Johns Hopkins University Department of Physical Medicine and Rehabilitation, Kessler Institute for Rehabilitation, Moss Rehabilitation Hospital, MetroHealth Rehabilitation Institute of Ohio, the Rehabilitation Institute of Chicago, and UDSMR.

The goal for the symposium was to serve as a catalyst for expanded research efforts on PAC rehabilitation so that relevant research can be used as the basis for policy and funding decisions. The planning committee sought to develop an agenda for research that supports an evidence base for PAC rehabilitation, including issues related to measurement and research design, access to PAC rehabilitation services, organization of rehabilitation services, and outcomes attained for beneficiaries of Medicare and other insurers. The objectives were to: (1) describe the current state of our knowledge regarding utilization, organization and outcomes of postacute rehabilitation settings; (2) identify methodologic and measurement challenges to conducting research in this area; (3) foster the exchange of ideas among researchers, policy-makers, industry representatives, funding agency staff, consumers, and members of advocacy groups; and (4) identify critical questions related to setting, delivery, payment, and effectiveness of rehabilitation services that are of the highest priority for investigation.

The activities of the symposium were designed to help formulate a research and policy agenda and to stimulate policy discussions, to engage stakeholders who are involved in policy decisions, and to provide emphasis for the need for an evidence base for rational policymaking. Symposium organizers sought balance in perspectives of key stakeholders, including Congress, the CMS and private insurers, providers of rehabilitation services, patients and their advocates, and health service researchers.

The planning committee invited research and policy leaders to present plenary and track-specific state-of-the-science summary speakers, and rehabilitation researchers to provide reports on contemporary work funded by AMRPA, the Rehabilitation Research and Training Center and other agencies. The planning committee invited 3 keynote speakers, former Senator Robert Dole; Laurence Wilson, director, Chronic Care Policy Group, CMS; and Steven Tingus, director, NIDRR. Four plenary speakers were invited to address each of the track themes. Articles by Pamela Duncan and Craig Velozo [3] (on measurement and methods), Melinda Beeuwkes Buntin [4](on access), Sally Kaplan [5] (on service organization), and Robert Kane [6] (on effectiveness) in this series were developed for the symposium. Four articles were commissioned to summarize the state-of-the-science and to provide commentary on the 24 work-in-progress presentations made at the symposium. Authors were Mark Johnston et al [7] (on measurement and methods), Ken Ottenbacher and James E. Graham [8] (on access), Leighton Chan [9] (on service organization), and Janet Prvu Bettger and Margaret Stineman [10] (on effectiveness).

More than 270 participants represented 166 organizations, including the U.S. Congress, CMS, NIDRR, NCMRR, private insurers, providers of rehabilitation services, patients and their advocates, and health researchers located primarily in academic institutions. They attended presentations by 3 keynote speakers, 4 plenary speakers, and concurrent breakout presentations organized by track theme. In addition, 20 peer-reviewed poster presentations summarized recently completed research.

The 4 concurrent breakout sessions, which were facilitated by assigned leaders and reporters, included 24 work-in-progress presentations and 4 state-of-the-science summaries by leading researchers, followed by roundtable discussions. These discussions were used to help assure that all participants had input to the process. Discussion leaders explained that the purpose of the discussion was to generate a report to the whole group that identified problems and solutions within the specific topic. Each breakout group then formulated research recommendations designed to improve our knowledge of how to organize and deliver effective rehabilitation services.

On the second day of the symposium, Barbara Gage, PhD [11], the principal investigator on the Deficit Reduction Act of 2005's Post Acute Care Demonstration project, described work underway to develop a common patient assessment instrument and study PAC payment reform for CMS.

Work groups developed recommendations for future research, and reviewed their recommendations during a general session. The reporters (Patrick Murray, Dexanne Clohan, Joy Hammel, Elizabeth Durkin) and discussion leaders (Bruce Gans, Greg Worsowicz, Dan Graves, John Whyte) summarized the recommendations which appear as the final report in the series. [12]

The remainder of this summary encapsulates key points from the plenary and state-of-the-science presentations followed by the track-specific research recommendations.

### Measurement and methodology

Patient assessment data are collected in 3 of the 4 PAC settings. SNFs use an instrument called the Minimum Data Set 2.0, HHAs use the Outcome and Assessment Information Set, and IRFs use the Inpatient Rehabilitation Facility Patient Assessment Instrument, which includes the FIM instrument. LTCHs do not have a mandate to use an assessment instrument. Although these instruments include similar items, the item definitions and assessment periods are different. Further, for the functional assessment domain, all 3 instruments were designed with a fixed set of items, regardless of relevance. In their plenary session, Duncan and Velozo [3] called for the development of clinical measures that are precise and sensitive to change across a wide range of patients, are retrievable in electronic medical records, and assess clinically relevant outcomes. Johnston et al [7] called for a method of grading the strength of evidence for and validity of PAC measures. Evidence is needed to support measures' content validity, reliability, internal structure validity, sensitivity to change, and predictive validity for outcomes or decisions (criterion-oriented validity). The development of a common assessment instrument across PAC providers will facilitate research, but issues regarding the timing of data collection may remain, because treatment phases intersect at varying points with a patient's recovery trajectory. Measurement of rehabilitation interventions was regarded as a major topic and was acknowledged to be the "weakest leg of the stool," whether the focus is specific treatment content or measures of organizational structure, process, or communication. Participants expressed an urgent need to develop validated measures that would allow rehabilitation to be judged.

Research priorities suggested by the measurement and methodology track participants included: develop validated measures of rehabilitation treatments; develop stronger cognitive and psychosocial outcome measures; develop long-term outcomes measures; develop robust severity and selection adjusters across the PAC rehabilitation patient population; assess the role of environmental factors on patient outcomes; and continue development of evidence-based treatment guidelines.

### PAC Access

Buntin [4] identified key concerns related to PAC access, including reduced access to care for complex cases, receipt of inappropriately low intensities of care, premature discharges, and receipt of care that may be unnecessary. Yet, there is a lack of clear evidence about which provider and treatment intensities are appropriate for specific patients. A few studies have examined use of PAC for patients with hip fracture and stroke. They found wide variation in utilization across geographic regions, which likely reflect practice styles, the supply of services, local practice regulations and substitution of services across sites. Ottenbacher and Graham [8] suggested that potential indicators of access to rehabilitation services may be classified into 4 types of barriers, including financial, structural, personal and sociodemographic, and attitudinal. This framework may be used to monitor access to PAC rehabilitation services.

Research priorities related to access include projecting the PAC needs of the population and determining the range and geographic distribution of existing PAC entities. Research should be directed to understand better how access is influenced by attitudes about family dynamics, social support, and cultural differences, as well as assumptions about the value of improvement for a patient who will not achieve complete independence.

### Care processes across PAC

Kaplan [5] described how MedPAC uses 6 indicators to assess payment adequacy for the 4 PAC sectors. The indicators are beneficiaries' access to care; supply of providers; utilization volume; quality; and providers' access to capital; and payment and costs. In 2006, all indicators suggested adequate payments for all 4 sectors. However, in 2007, all indicators suggested adequate payment for HHAs; all indicators but quality were favorable for SNFs; all LTCH indicators were favorable, except there was a drop in the Medicare margin from 2005; and IRF indicators were mixed.

Chan [9] described how postacute rehabilitation care is fragmented into 4 "silos" based on provider type. This lack of integration provides disincentives for delivering the most cost-effective sequence of postacute services. Each provider type has a unique Medicare payment system with unique incentives. For example, SNFs and HHAs have strong incentives to provide rehabilitation services, while IRFs and LTCHs have incentives to reduce their average length of stay. Little policy research has been reported about the impact of Medicare's payment systems on PAC services overall and these policies continue to evolve. The goal in PAC should be to provide the right "dose" of care to the right patient at the right time in the right place.

Participants in the processes of care track suggested that future research include randomized trials that test individual components of PAC care to determine optimal intensity, duration, and frequency of interventions. To overcome the current barriers of conducting research across provider types, the experiences of other health care systems such as the Veteran's Administration and Kaiser Permanente should be examined.

### PAC rehabilitation effectiveness

Kane [6] discussed a number of issues related to the effectiveness of PAC, including outcomes that are a function of baseline status, patient clinical characteristics, demographic characteristics, and treatments. He also contrasted pay-for-performance systems based on process indicators (eg, guideline adherence) with case-mix adjusted outcome, and argued that we should encourage rehabilitation activities that have been shown to yield improvements in quality-adjusted life years. Prvu Bettger and Stineman [10] described how randomized controlled trials are not appropriate for investigating all areas of rehabilitation, but that well-designed nonrandomized trials can advance our knowledge base. The Transparent Reporting of Evaluations with Non-randomized Designs statement may help improve the quality of effectiveness research. They recommended that well-designed, nonrandomized trials should be used to complement randomized trials to study real-world clinical practice.

Participants in the effectiveness group suggested that future research should focus on what kind of treatment, or combination of services, is most effective in achieving specific outcomes for whom across the continuum of care. In addition, better measures of PAC rehabilitation treatments are needed so that key contents or treatments are identified and can be studied systematically and compared across the continuum of PAC. Participants expressed a strong need for a strategic research plan that is shared by payers, providers, research funders, and researchers; a common measurement time period; and collaboration between CMS, National Institutes of Health, the NIDRR, and the research community to provide flexibility within rigorously designed research protocols, because the PPS itself is a primary obstacle to treatment innovation.

### In memory of 2 rehabilitation research leaders

Two colleagues who made major contributions to research and policy discussions on rehabilitation outcomes were very much with symposium participants in spirit, though their recent passing leaves us with a major loss. Deborah Wilkerson and Robert Allen Keith made enormous contributions to rehabilitation research. Wilkerson was a researcher, administrator, and national leader on outcomes measurement, rehabilitation services quality, postacute payment policy, and independent living issues. She led the outcomes measurement and performance indicator programs at CARF and spearheaded the research and development for *uSPEQ: Giving Quality a Voice*, CARF International's customer feedback service. Keith began his affiliation with Casa Colina in the 1950s. He began volunteering as a clinical psychologist and soon developed a special interest in rehabilitation services, which eventually led him to develop Casa Colina's Research Department. He became a pioneer in the study of rehabilitation outcomes, published extensively, and helped contribute to the development of the industry standard method of assessing functional status. Our accomplishments are a reflection of their dedication and inspiration.

### Summary

Postacute rehabilitation care is a key component of the health care delivery system, yet we know little about the active ingredients of the rehabilitation process that produce the best outcomes. Well-designed research is needed to develop better measures for case-mix adjustment and outcomes of care. To advance rehabilitation effectiveness research and support the development of evidence-based policies, we must invest in developing new and improve existing measures of patient characteristics, treatment contents, and long-term outcomes. Critical research needs include (1) developing validated measures of rehabilitation interventions and case mix; (2) standardizing PAC measures and timing of routine measurement for payment and quality assurance purposes across sites of care; (3) examining differences in content and processes of care both within facilities of the same type and across types of facilities; (4) identifying patient characteristics that vary by region such as rural and urban mix, cultural characteristics, and provider referral patterns; and (4) implementing a "strategic plan for effectiveness research" that is characterized by collaboration between CMS, federal research funders, researchers, and care sites.

The organizers and sponsors of this symposium trust that our goal of catalyzing expanded research on PAC rehabilitation is furthered by the publication of this set of articles. Our nation's health policy requires a solid base founded on compelling evidence. We look forward to the benefits of greater research attention to improved measurement and research design, access to PAC rehabilitation services, organization of rehabilitation services, and outcomes attained for patients, taxpayers, and Medicare and other insurers.

The content developed for and derived from the symposium can be found in the November 2007 issue of *Archives of Physical Medicine and Rehabilitation* (additional symposium information is available at [13]).

## Competing interests

Supported by the National Institute on Disability and Rehabilitation Research through a Rehabilitation Research and Training Center on Measuring Rehabilitation Outcomes and Effectiveness (grant no. H133B040032).

No commercial party having a direct financial interest in the results of the research supporting this article has or will confer a benefit upon the author or upon any organization with which the author is associated.
